# Iron Release from Soybean Seed Ferritin Induced by Cinnamic Acid Derivatives

**DOI:** 10.3390/ph11020039

**Published:** 2018-05-04

**Authors:** Xuejiao Sha, Hai Chen, Jingsheng Zhang, Guanghua Zhao

**Affiliations:** Beijing Advanced Innovation Center for Food Nutrition and Human Health, College of Food Science and Nutritional Engineering, Beijing Key Laboratory of Functional Food from Plant Resources, China Agricultural University, Beijing 100083, China; cau3060509@163.com (X.S.); chenhai2509@163.com (H.C.); zhangjingsheng@cau.edu.cn (J.Z.)

**Keywords:** cinnamic acid derivatives, soybean seed ferritin, iron release, binding ability, Fe^2+^-chelating activity, reducibility

## Abstract

Plant ferritin represents a novel class of iron supplement, which widely co-exists with phenolic acids in a plant diet. However, there are few reports on the effect of these phenolic acids on function of ferritin. In this study, we demonstrated that cinnamic acid derivatives, as widely occurring phenolic acids, can induce iron release from holo soybean seed ferritin (SSF) in a structure-dependent manner. The ability of the iron release from SSF by five cinnamic acids follows the sequence of Cinnamic acid > Chlorogenic acid > Ferulic acid > *p*-Coumaric acid > *Trans*-Cinnamic acid. Fluorescence titration in conjunction with dialysis results showed that all of these five compounds have a similar, weak ability to bind with protein, suggesting that their protein-binding ability is not related to their iron release activity. In contrast, both Fe^2+^-chelating activity and reducibility of these cinnamic acid derivatives are in good agreement with their ability to induce iron release from ferritin. These studies indicate that cinnamic acid and its derivatives could have a negative effect on iron stability of holo soybean seed ferritin in diet, and the Fe^2+^-chelating activity and reducibility of cinnamic acid and its derivatives have strong relations to the iron release of soybean seed ferritin.

## 1. Introduction

**Figure 1:**
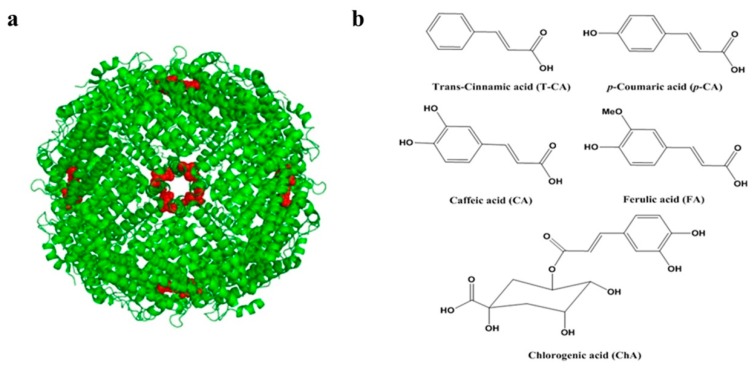
(**a**) Crystal structure of plant ferritin with views down the four-fold axes (channels) of the protein shell. The tryptophan residue of each subunit is highlighted in red; (**b**) Chemical structure of cinnamic acid derivatives: *Trans*-cinnamic acid (T-CA); *p*-Coumaric acid (*p*-CA); Caffiec acid (CA); Ferulic acid (FA); Chlorogenic acid (ChA).

Iron plays an essential role in living organisms, such as oxygen transfer, DNA synthesis, electron transport, and tricarboxylic acid and nitrogen fixation. Actually, iron deficiency is one of the most serious global nutritional problems, which affects about two billion people in the world [1]. Although the prevalence of iron deficiency anemia (IDA) is higher in developing countries, iron deficiency is still common among women and young children in industrial countries. More importantly, IDA can cause a series of consequences, such as reduced cognitive, motor development in infants and poor pregnancy outcomes. Ferritin is a natural and ubiquitous iron storage protein occurring widely in plants, animals, bacteria and fungi [2]. It consists of 24 subunits that assemble in a highly symmetric manner to a hollow protein shell with the outside diameter of about 12–13 nm and inner diameter of about 7–8 nm [3]. One ferritin molecule contains six four-fold, eight three-fold, and twelve two-fold channels (Figure 1a), which connect the inner cavity to bulk solution and serve as multiple pathways for iron entry and exit. Ferritin can store up to 4500 Fe^3+^ in its cavity, and therefore, natural ferritin, especially plant ferritin from legume seeds, has been considered as a novel alternative dietary iron supplement against IDA [1]. As compared to other iron supplements with a small size, ferritin has three major advantages: the protection of protein shell from interaction with other dietary factors, the safer form of iron stored as ferric cores rather than ferrous iron, and the possible intact absorption by receptor-mediated endocytosis [1]. 

So far, Fe^2+^ oxidation and mineral deposition in ferritin have been studied intensively and extensively [1,4]. Although some reductants such as ascorbate, 6-hydroxydopamine, 5-aminolevulinic acid, superoxide anion radical, 1,2,4-benzenetriol, benzene metabolites, NADH and anthocyanins have been shown to induce iron release from ferritin [5,6], and several iron chelators including 2, 2′-bipyridine, salicylate, citrate, nitrilotriacetate, and desferroxamine B can also induce iron release from ferritin at a slower rate [7,8,9]. However, there has been less attention paid to the iron release of ferritin induced by nutrients and the stability of ferritin iron core in food systems.

Phenolic acids are secondary metabolites, which are widely distributed in various plant foodstuffs. Among these phenolic acids, cinnamic acid derivatives (Figure 1b), such as caffeic, chlorogenic, *p*-coumaric and ferulic acids, are reported to possess functional activities like cancer prevention, antituberculosis, antileukaemic, hepatoprotective, antidiabetic, antioxidative, and hypocholesterolemic activities [10,11,12]. However, to date, whether these phenolic acids have an effect on the function of protein through intermolecular interaction has received much less attention. Based on the fact that phenolic acids such as cinnamic acid derivatives and ferritin co-exist in plant foodstuffs, and that cinnamic acid derivatives have a strong reducing activity, while ferric iron within phytoferritin exhibits a relatively strong oxidative activity, it is of special interest to know if the two kinds of molecules could interact with each other, and if so, what is the consequence of such interaction, which is the focus of this work.

## 2. Results and Discussion

### 2.1. Isolation and Characterization of SSF and rH-2

Ferritin can stay in two forms, holo and apo ferritin. Naturally occurring ferritin is a holo form which contains hydrous ferric oxide nanoparticles as iron cores within its cavity. Holo plant ferritin usually consists of two H type subunits of 26.5 (H-1) and 28.0 kDa (H-2), while their ratios vary among species. In this study, natural holo soybean seed ferritin (SSF) was used for iron release experiments because it is most extensively studied among all known plant ferritins [1,13]. However, our recent studies have showed that SSF is unstable during storage, because the extension peptide (EP) of the H-1 subunit exhibits significant serine protease-like activity, which is located at the N-terminal extremity. Consequently, recombinant soybean seed H-1 ferritin (rH-1) is prone to degradation, whereas its analogue, recombinant soybean seed H-1 ferritin (rH-2), becomes very stable under identical conditions [14]. Therefore, apo rH-2 was used to study the interaction between cinnamic acid derivatives and protein. 

**Figure 2:**
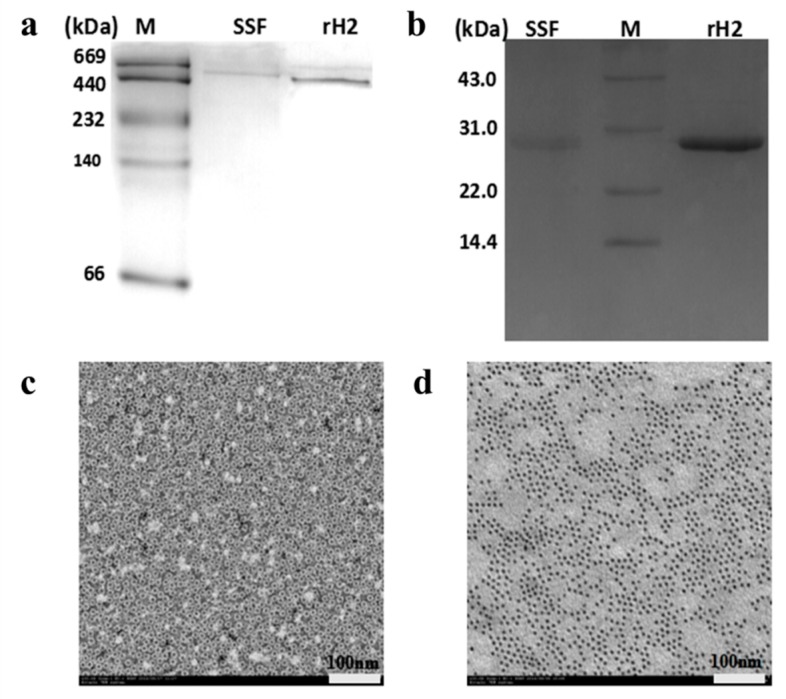
(**a**) Native PAGE analyses and (**b**) SDS-PAGE of soybean seed ferritin (SSF) and recombinant H2 ferritin (rH-2); (**c**) TEM picture of purified rH-2 after beingnegatively stained by 2% uranyl acetate; (**d**) TEM picture of holo soybean seed ferritin (SSF) without negative staining of uranyl acetate.

After purification, these two kinds of ferritins were analyzed by PAGE and TEM. Nondenaturing gel electrophoresis (native PAGE) revealed the purified SSF and rH-2 as a single complex (Figure 2a), suggesting that they have been purified to homogeneity. SDS-PAGE indicated that SSF consists of nearly 28.0 and 26.5 kD subunits, a result consistent with previous observations [15]. As expected, rH-2 was composed of only one 28.0 kD subunit (Figure 2b). TEM analyses revealed that rH-2 molecules were well dispersed with an outside diameter of ~12 nm (Figure 2c) after being negatively stained with uranyl acitate. In contrast, without negative staining, iron cores can be clearly observed in holo SSF (Figure 2d) as isolates contains ~1800 g atom of iron [1].

### 2.2. Iron Release from SSF Induced by Cinnamic Acid Derivatives

**Figure 3:**
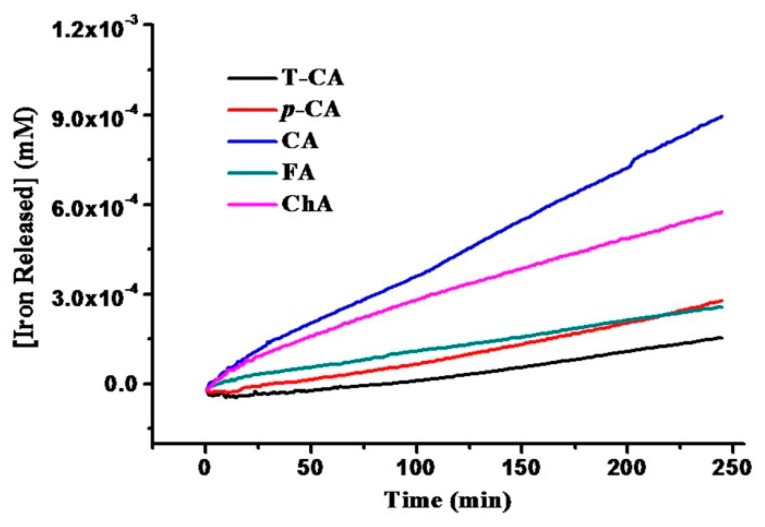
Kinetics of iron release from holo SSF induced by cinnamic acid derivatives. Iron release from SSF in the presence of reductants was followed by measuring the increase in absorbance at 562 nm about 250 min due to the chelation of Fe^2+^ by ferrozine. Conditions: 0.15 µM SSF, 25 μM cinnamic acids, 50 mM Mops, pH 7.0, 0.15 M NaCl, 500 μM ferrozine, 25 °C.

Subsequently, we analyzed the possibility of all cinnamic acid derivatives to induce iron release from SSF at a concentration of 25 µM [5], and results are displayed in Figure 3. It was found that all of them not only were able to cause iron release from protein shell, but also such iron release showed a structure-dependent manner. For example, CA induces iron release from SSF at the initial rate of 0.104 ± 0.006 mM/min, which is fastest among all tested compounds. The rate of iron release follows the sequence CA > ChA > FA > *p*-CA > T-CA in Mops buffer (pH 7.0) at 25 °C. CA has the higher ability to induce iron release from ferritin as compared to its analogue, ChA (0.092 ± 0.021) mM/min. This might be derived from the fact that ChA contains a sinapic acid group in its structure, thereby its large size prevents it from diffusion into the protein shell to some extent. These results suggest that the rate of iron release is inversely proportional to the size of phenolic acids, which is in agreement with a previous report [16]. Thus, it seems that the molecular size of phenolic acids plays an important role during the iron release from SSF induced by reductants. 

Besides, it was observed that the number of HO groups of cinnamic acid derivatives has a marked effect on the rate of iron release from ferritin. For example, with an increase in the number of HO from 0, 1, to 2, the rate of the iron release from ferritin increased from 0.002 ± 0.001 mM/min for T-CA, 0.014 ± 0.004 mM/min for *p*-CA, to 0.104 ± 0.006 mM/min for CA, respectively, suggesting that the number of HO group in cinnamic acid derivatives is closely associated with their ability to induce iron release from ferritin. Consistent with this idea, the rate of iron release from ferritin induced by CA was greatly decreased by ~50% after one HO group was replaced by OMe in FA (0.046 ± 0.011 mM/min) as shown in Figure 3. 

### 2.3. Fluorescence Quenching Analyses 

**Figure 4:**
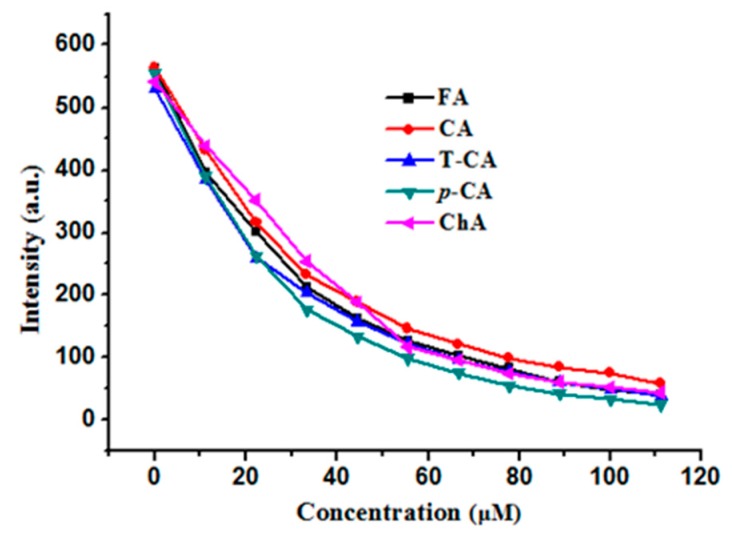
Comparison of cinnamic acids on the fluorescence quenching of apo rH-2. Conditions: 0.55 μM rH-2 in 50 mM Tris-HCl, [phenolic acids] = 0–110 μM, pH 7.0, 25 °C. λEx = 280 nm, slits for excitation and emission are 5 nm and 10 nm.

The above results demonstrated that all of these cinnamic acid derivatives are able to facilitate iron release from ferritin to different extents. To shed light on the mechanism by which iron release from ferritin was induced by these phenolic acids, we investigated the binding activity of these small molecules to ferritin through their interaction with protein. Protein intrinsic fluorescence mainly from Trp residues has been widely used to study their interaction with small molecules because of its sensitivity to microenviroment surrounding the fluorophore residue. As shown in Figure 1a, there are 24 Trp residues in one soybean seed ferritin molecule [17]. By taking advantage of this, we used intrinsic emission spectroscopy to study the interaction between these small molecules and apo rH-2, and results were shown in Figure 4. It was observed that all of cinnamic acid and its derivatives were able to quench the protein fluorescence, showing similar fluorescence quenching curves, indicative of similar interaction between cinnamic acid derivatives and apo rH-2. Thus, it seems that their interaction with apoferritin is independent of chemical structure, inconsistent with the above iron release results. 

**Figure 5:**
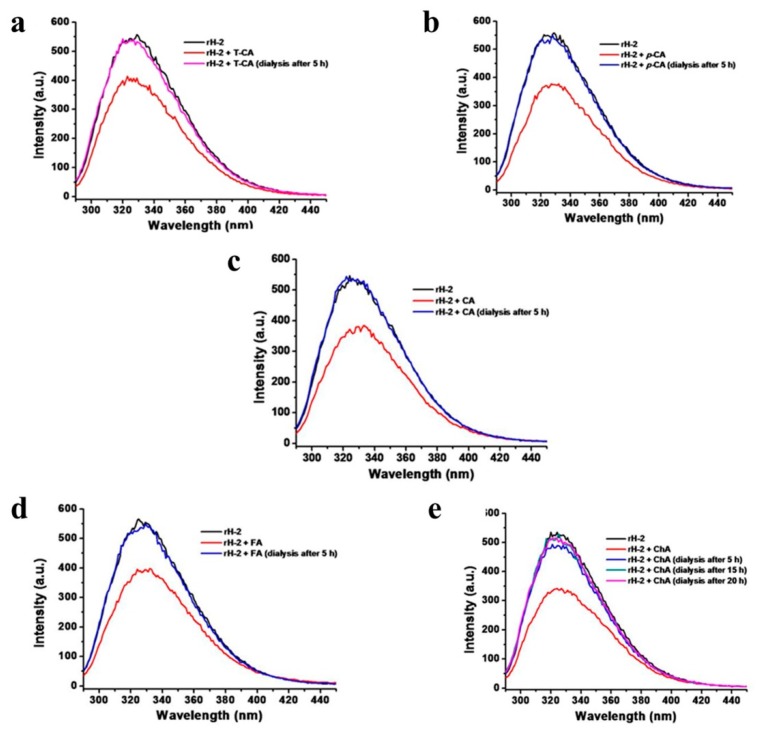
Dialysis study of rH-2 with cinnamic acids at pH 7.0. (**a**) *Trans*-Cinnamic acid; (**b**) *p*-Coumaric acid; (**c**) Caffeic acid; (**d**) Ferulic acid; (**e**) chlorogenic acid. Conditions: 0.55 μM rH-2 in 50 mM Tris-HCl, [cinnamic acids] = 22 μM, 25 °C. λ_Ex_ = 280 nm, slits for excitation and emission are 5 nm and 10 nm.

To determine whether these cinnamic acid derivatives can interact with proteins through binding, dialysis experiments were carried out wherein apo rH-2 (0.55 μM) was firstly incubated with these phenolic acids (22 μM), followed by dialysis against buffer for four times to remove free small molecules, respectively, and results are given in Figure 5. It was found that after dialysis for 5 h, the fluorescence of apo rH-2 could return to an original state upon treatment with these phenolic compounds, respectively (Figure 5). These results indicated that the fluorescence quenching of apo rH-2 caused by these compounds is dynamic, resulting from collisional encounters between the fluorophore and quencher. Instead, it is difficult to form a complex between ferritin and each of these cinnamic acid derivatives under the present experimental conditions; at most, the binding of the small compounds to apo rH-2 is very weak, and therefore, dialysis can remove them from their mixture with apo rH-2.

The weak binding activity of the cinnamic acid derivatives to ferritin might be derived from the fact that they contain less than three hydroxyl groups in the structure. Consistent with this view, our recent study showed that gallic acid, methyl gallate and propyl gallate having three HO groups can bind to rH-2 tightly, while their analogues with two HO groups cannot [18]. Thus, the number of the hydroxyl groups is closely associated with the interaction mode between phenolic acids and protein, and more hydroxyl groups in the structure favors the binding of phenolic acids to protein. Further support for this idea comes from a recent study showing that tannic acid with many hydroxyl groups can facilitate ferritn association through strong hydrogen bonds [19]. 

### 2.4. Effect of the Fe^2+^ Chelating Activity of Cinnamic Acid Derivatives on the Iron Release from Ferritin

**Figure 6:**
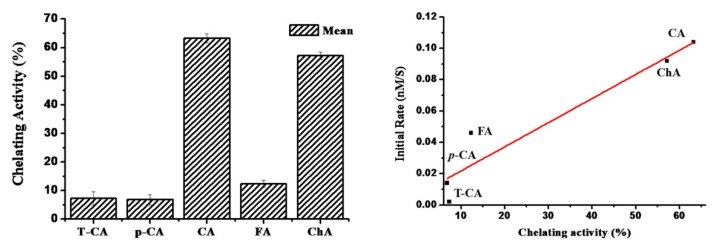
Chelating activity of cinnamic acid derivatives on ferrous ion. Vertical bars represent the standard error from means of three separate tests.

It was previously reported that the chelating activity of reductants on iron ion was an important factor which is related to iron release from the ferritin shell [20]. Therefore, in this study, the iron chelating activity of cinnamic acid derivatives was determined, respectively, to gain insight into the mechanism of iron release from ferritin. As shown in Figure 6a, the chelating activity of cinnamic acid derivatives on ferrous ions follows the sequence of CA > ChA > FA > T-CA ≈ *p*-CA. The chelating activity of CA and ChA with catechol moiety was ~63% and 58%, respectively, which was almost six-fold stronger than their analogue, FA (~11%) where one hydroxyl group was replaced with OMe. The large difference in the chelating activity between CA/ChA and FA suggested that two hydroxyl groups in the benzene ring of CA and ChA were required for Fe^2+^ chelating, while one hydroxyl group in the benzene ring of FA is not enough to chelate iron. Agreeing with this idea, both T-CA (~7.0%) and *p*-CA (~6.5%) with zero or one hydroxyl group exhibited even much weaker chelating activity than CA or ChA. Additionally, it was found that CA and ChA exhibited nearly the same chelating activity, suggesting that the carboxyl group might not be involved in iron chelating, and at most it contributed much less to iron chelating as compared to the hydroxyl group. 

More importantly, it was observed that the chelating activity of these phenolic acids was in good agreement with the above iron release results (Figure 3); namely, the stronger the chelating activity, the faster rate of the iron release from ferritin. Consistent with this conclusion, there is a linear relationship between the rate of iron release from ferritin induced by these phenolic acids and their chelating activity as shown in Figure 6b. Based on these findings, it is reasonable to believe that the chelating activity of cinnamic acid derivatives has an important effect on the rate of iron release from ferritin induced by these compounds. This might be because these cinnamic acid derivatives can act as iron chelators to help iron to move out of SSF. 

### 2.5. Effect of Reducibility of Cinnamic Acid Derivatives on the Iron Release from Ferritin

**Figure 7:**
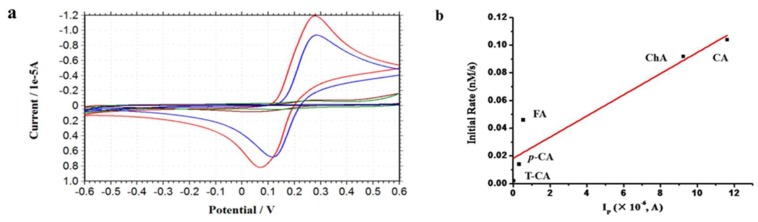
Cyclic voltammograms taken with a 3-mm glassy carbon electrode at 100 mVs^−1^ of: 0.5 mM cinnamic acid (mazarine line), *p*-coumaric acid (green line), caffeic acid (red line), ferulic acid (carmine red), and chlorogenic acid (blue red) in 50 mM phosphate buffer of pH 7.0.

To better understand the mechanism of iron release from ferritin, we also studied the voltammetric oxidation of these phenolic acids in pH 7.0 phosphate buffer by cyclic voltammetry, and results are shown in Figure 7a. Cyclic voltammograms of CA, FA, *p*-CA and ChA at a sweep rate of 100 mM s^−1^ exhibited one anodic peak and one cathodic peak, suggesting that the oxidation process for these four investigated compounds was reversible. However, cyclic voltammograms of T-CA showed no peak, indicating that T-CA has no reducibility [21]. As shown Table 1, ChA (0.31 V) has the largest anodic peak potential among all of the five compounds.

Interestingly, the oxidation peak current of the cinnamic acid derivatives follows the sequence of CA > ChA > FA > *p*-CA > T-CA (Table 1). This trend is in good agreement with the initial rate of iron release from SSF induced by these compounds. For reversible systems, the oxidation peak current, I_p_ could represent the reducibility of the analyte in the solution bulk [22]. Caffeic acid and chlorogenic acid with two hydroxyl groups in their benzene ring structure show the stronger oxidation peak current as compared with chlorogenic acid. In contrast, FA and *p*-CA with only one hydroxyl group have weaker oxidation peak current, while T-CA with no HO group has no reducibility. These results indicated that the reducibility of cinnamic acids can greatly influence the rate of iron release from SSF. Indeed, there is also a linear relationship between the oxidation peak current of the cinnamic acid derivatives and their ability to induce iron release from ferritin as shown in Figure 7b. Therefore, the reducibility of phenolic acids also is another important factor affecting their ability to induce iron release from the ferritin shell. 

## 3. Materials and Methods

### 3.1. Chemicals

Five cinnamic acid derivatives used in this study were purchased from J&K Chemical (Beijing, China). Ferrous ion chelator 3-(2-pyridyl)-5,6-bis(4-phenylsulfonic acid)-1,2,4-triazine (ferrozine) was obtained from Sigma-Aldrich Chemical Co. (Beijing, China). Sephacryl S-300, DEAE Sepharose Fast Flow, native electrophoresis marker, and SDS electrophoresis marker were purchased from GE Healthcare Bio-Sciences AB (Beijing, China). Sodium citrate, terephthalic acid (TA) and magnesium chloride hexahydrate were obtained from Beijing Chemical Reagents Co. (Beijing, China). All other reagents used were of analytical grade or purer.

### 3.2. Preparation of Soybean Seed Ferritin (SSF) and Recombinant Soybean Seed H-2 Ferritin (rH-2)

SSF and apo rH-2 were purified as previously described with some modification [23,24]. Typically, approximately 1 kg of soybean seeds was soaked in distilled water for 8 h and mixed with three volumes of extraction buffer (50 mM KH_2_PO_4_, pH 7.5, 1% polyvinylpolypyrrolidone). After filtration, the filtrate was incubated for 15 min at 60 °C and then was centrifuged at 5000× *g* for 5 min to separate the insoluble material. The supernatant was adjusted to 0.5 M MgCl_2_ and the mixture stood for 30 min at 4 °C followed by addition of sodium citrate (final concentration of 0.7 M) to complex the magnesium. After 10 h, the resultant supernatant was centrifuged at 12,000× *g* for 20 min at 4 °C. The brown precipitate thus obtained was dissolved in 50 mM KH_2_PO_4_•Na_2_HPO_4_ buffer (pH 7.5) and was dialyzed against the same buffer three times. 

*Escherichis coli* strain BL21 (DE3) which contained rH-2 expression plasmids was grown at 37 °C. Protein expression was induced with 1 mM IPTG (isopropyl β-D-1-thiogalactopyranoside) for 7 h. The system was centrifuged and re-suspended in buffer solution (50 mM Tris-HCl, pH 8.0), followed by disruption by sonication. The supernatant of the crude extract was collected by centrifugation and further purified by ammonium sulfate fractionation (40% saturated fraction). After washed by distilled water to remove other proteins, the sediment was dissolved by solution buffer. Next the solution was dialyzed against the same buffer three times to remove the ammonium sulfate. SSF and rH-2 protein were further purified by Sephacryl S-300 gel filtration chromatography (GFC) and DEAE-Sepharose Fast Flow column, respectively. Finally, protein purity was analyzed by SDS-PAGE and Native-PAGE analysis. Protein concentrations were determined according to the Lowry method as previous reported [25]. 

### 3.3. Kinetic Measurement of Iron Release from Holo Soybean Seed Ferritin

The assay system (1 mL of total volume) contained 0.21 μM ferritin, 500 μM ferrozine, 0.15 M NaCl, and 25 μM cinnamic acid derivatives in 50 mM Mops buffer, pH 7.0 as previously described [5]. The same solution but just using buffer instead of cinnamic acid derivatives was used as control. Experiments were conducted at 25 °C. The iron release was measured with the development of [Fe(ferrozine)_3_]^2+^ at 562 nm using a Varian Cary 50 spectrophotometer, and ε_562_ = 27.9 mM^−1^cm^−1^. The absorbance at 562 nm from the control was deducted from that of all sample mixtures. The initial rate (*v*_0_) of iron release measured as [Fe(ferrozine)_3_]^2+^ formation was obtained from the linear *A*_1_ term of third-order polynomial fitted to the experimental data as reported previously: *Y* = *A*_0_ + *A*_1_t + *A*_2_t^2^ + *A*_3_t^3^ and d*Y*/dt = *A*_1_ + 2*A*_2_t + 3*A*_3_t^2^ (at t = 0, (d*Y*/dt)_0_ = *v*_0_). Here t is the time in minutes and *Y* is the concentration of [Fe(ferrozine)_3_]^2+^ at time t in minutes [16].

### 3.4. Fluorescence Titration Analysis 

Fluorescence titration measurements were recorded on a Cary Eclipse fluorescence spectrophotometer (Varian, Polo Alto, CA, USA), using quartz cuvettes of 1 cm path length at room temperature. In this measurement, apo rH-2 (0.55 μM, 150 mM NaCl and 50 mM Tris-HCl, pH 7.0) was titrated with 1 μL cinnamic acid derivatives (5 mM, 50 mM Tris-HCl, pH 7.0), respectively. The excitation wavelength was 280 nm, and the emission wavelength was 325 nm. Furthermore, dialysis measurements were conducted to analyze the binding affinity of cinnamic acids to apo rH-2. Typically, a mixture of apo rH-2 (0.55 µM) plus cinnamic acid derivatives (22 µM) was dialyzed (100 kDa cutoff) for 5 h against the solution buffer (50 mM Tris-HCl and 150 mM NaCl, at pH 7.0) to detect this kind of interaction [18].

### 3.5. Chelating Activity of Cinnamic Acid Derivatives on Ferrous Ion

The chelating activity of cinnamic acid derivatives on ferrous ion was measured as described previously with some modifications [5]. The reaction system (1 mL) contained 20 µM FeSO_4_, 60 µM ferrozine, and 60 µM cinnamic acid derivatives in 50 mM Mops buffer (pH 7.0). After reaction for 5 min at 25 °C, the absorbance of system was detected at 562 nm spectrophotometrically. The ability of cinnamic acids to chelate Fe^2+^ was calculated as follows: chelating activity (%) = $\frac{A0-A}{A0}$ × 100, where *A* and *A*_0_ are the absorbance in the presence or absence of the cinnamic acid derivatives, respectively.

### 3.6. Cyclic Voltammetry 

Cyclic voltammetry experiments were performed using a potentiostat (microAutolab Type III with an Autolab Faraday Cage) and voltammograms were obtained with a scan rate of 100 mV s^−1^ with an increment potential of 2.4 mV, between −0.6 V and 0.6 V. The working electrode was a 3 mm glassy carbon disk in combination with a Metrohm tipholder, cleaned by polishing with 3 µm alumina powder during 30 s followed by fixing the potential in ultrasonication during 5 s, between acquisitions. A saturated calomel electrode was used as a reference electrode in conjunction with a platinum counter electrode. Each acquisition required 40 mL of sample. Oxygen was removed with a N_2_ current flow during 5 min prior to analysis. Cyclic voltammetry experiments were controlled by the CHI600E Electrochemical Workstation. Cyclic voltammograms were taken in the absence and in the presence of cinnamic acid derivatives.

### 3.7. Statistical Analysis

All data analyses were performed using Origin 8.0 software and the structural formula was processed by ChemDraw 7.0. All experiments were carried out in triplicate.

## 4. Conclusions

The present study demonstrates that cinnamic acid derivatives, a class of naturally occurring phenolic acids, can induce iron release from holo soybean seed ferritin (SSF) in a structure-dependent manner for the first time. The ability of iron release from SSF by five cinnamic acids follows the sequence of CA > ChA > FA > *p*-CA > T-CA. Although the five phenolic acids exhibit a weak ability to bind with ferritin, they are able to induce iron release from holo ferritin through their Fe^2+^-chelating activity and reducibility. These studies indicate that cinnamic acid and its derivatives could have a negative effect on iron absorption in humans, because they can result in loss of a certain amount of iron from holo ferritin through interactions. Therefore, the interactions between cinnamic acid derivatives and holo SSF should be avoided as much as possible during food processing.

## Figures and Tables

**Table 1:** Evaluation of the anodic peak potentials of the studied cinnamic acid derivatives (E_p_—anodic peak potential (V)) at pH 7.0, as well as the anodic peak currents at pH 7.0 (I_P_—anodic peak current (A)) during the cyclic voltammetry experiment.

Phenolic Compound	E_p_	I_p_ (×10^−^^6^)
T-CA	0	0
*p*-CA	0.27	0.31 ± 0.12
CA	0.28	11.61 ± 0.35
FA	0.24	0.53 ± 0.07
ChA	0.31	9.23 ± 0.46
